# Supplementary material for: Structure, Function and Dynamics of mCoral, a pH-Responsive Engineered Variant of the mCherry Fluorescent Protein with Improved Hydrogen Peroxide Tolerance
Source: Int J Mol Sci. 2025 Dec 23;27(1):154. doi: 10.3390/ijms27010154 (PMC12785414; doi:10.3390/ijms27010154)
Supplement: Supplementary file 1 [file ijms-27-00154-s001.zip › ijms-4038670-supplementary.pdf]

Structure, function and dynamics of mCoral, a pH responsive engineered variant of the mCherry fluorescent protein with improved hydrogen peroxide tolerance.

Athena Zitti[1], Ozan Aksakal[1], Danoo Vitsupakorn[1], Pierre J. Rizkallah[2], Halina Mikolajek[3], James A. Platts[4], Georgina E. Menzies[1], D. Dafydd Jones[1]*

1. Molecular Bioscience Division, School of Biosciences, Cardiff University, Cardiff, UK
2. School of Medicine, Cardiff University, Cardiff, UK
3. Diamond Light Source, Harwell, UK.
4. School of Chemistry, Cardiff University, Cardiff, UK.

* Corresponding author: D. Dafydd Jones, School of Biosciences, Sir Martin Evans Building, Cardiff University, Cardiff, CF10 3AX, UK. Email: jonesdd@cardiff.ac.uk. Telephone: +44 2920874290.

Supplementary Information.

Supplementary Table S1. Data collection and refinement statistics for mCoral

**PDB Entry**	
**Data Collection***	
Diamond Beamline	I03
Date	2021-09-30
Wavelength	0.81532
**Crystal Data (figures in brackets refer to outer resolution shell)**	
a,b,c (Å)	48.760, 43.182, 63.095
α,β,γ (°)	90.0, 114.91, 90.0
Space group	$P\ 1\ 2_1\ 2$
Resolution (Å)	2.04 – 45.46
Outer shell	2.04 – 2.10
R-merge (%)	9.1 (46.7)
R-pim (%)	9.1 (46.7)
R-meas (%)	12.8 (66.1)
CC1/2	0.980 (0.413)
I / σ(I)	20.5 (4.7)
Completeness (%)	97.3 (98.3)
Multiplicity	1.8 (1.8)
Total Measurements	27,152 (2,107)
Unique Reflections	14,902 (1,149)
Wilson B-factor($Å^2$)	20.2
**Refinement Statistics**	
Refined atoms	1,853
Protein atoms	1,761
Non-protein atoms	5
Water molecules	87
R-work reflections	13,309
R-free reflections	1,376
R-work/R-free (%)	16.98 / 21.06
**rms deviations (ML target in brackets)**	
Bond lengths (Å)	0.012 (0.013)
Bond Angles (°)	1.553 (1.661)
[1]Coordinate error	0.111
Mean B value ($Å^2$)	23.4
**Ramachandran Statistics (PDB Validation)**	
Favoured/allowed/Outliers	208 / 9 / 0
%	95.9 / 4.1 / 0.0

* One crystal was used for determining each structure.
[1] Coordinate Estimated Standard Uncertainty in (Å), calculated based on maximum likelihood statistics.

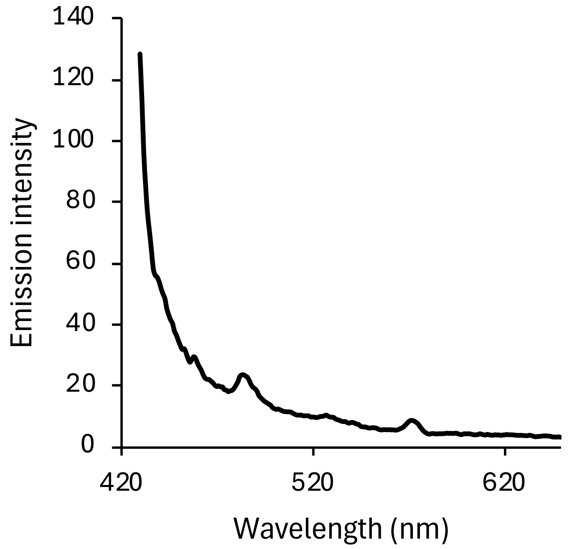

Supplementary Figure S1. The emission spectra of mCoral at pH 4.5 on excitation at the λ_{max} for that pH (420 nm).

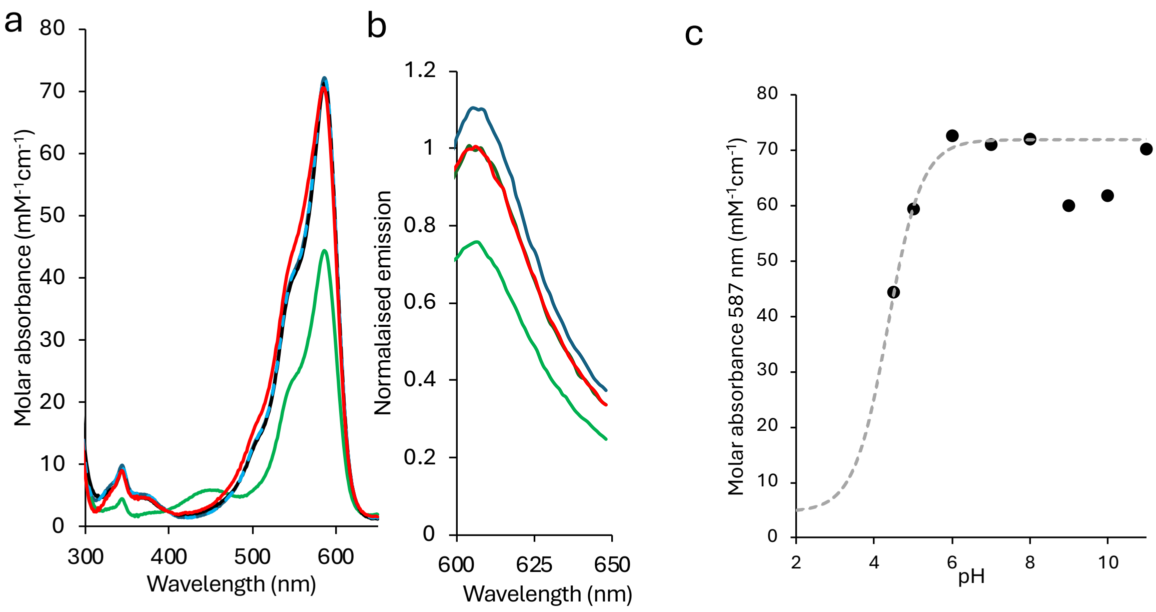

Supplementary Figure S2. The effect of pH on the spectral properties of mCherry. Change in (a) absorbance spectra and (b) emission spectra (on excitation at λ_{max}) at pH 4.5 (green), pH 7 (black), pH 8 (dashed blue line) and pH 11 (red). (c) Plot of change in 587 nm molar absorbance against pH fitted to a single transition sigmoidal curve according to the Henderson-Hasselbach equation.

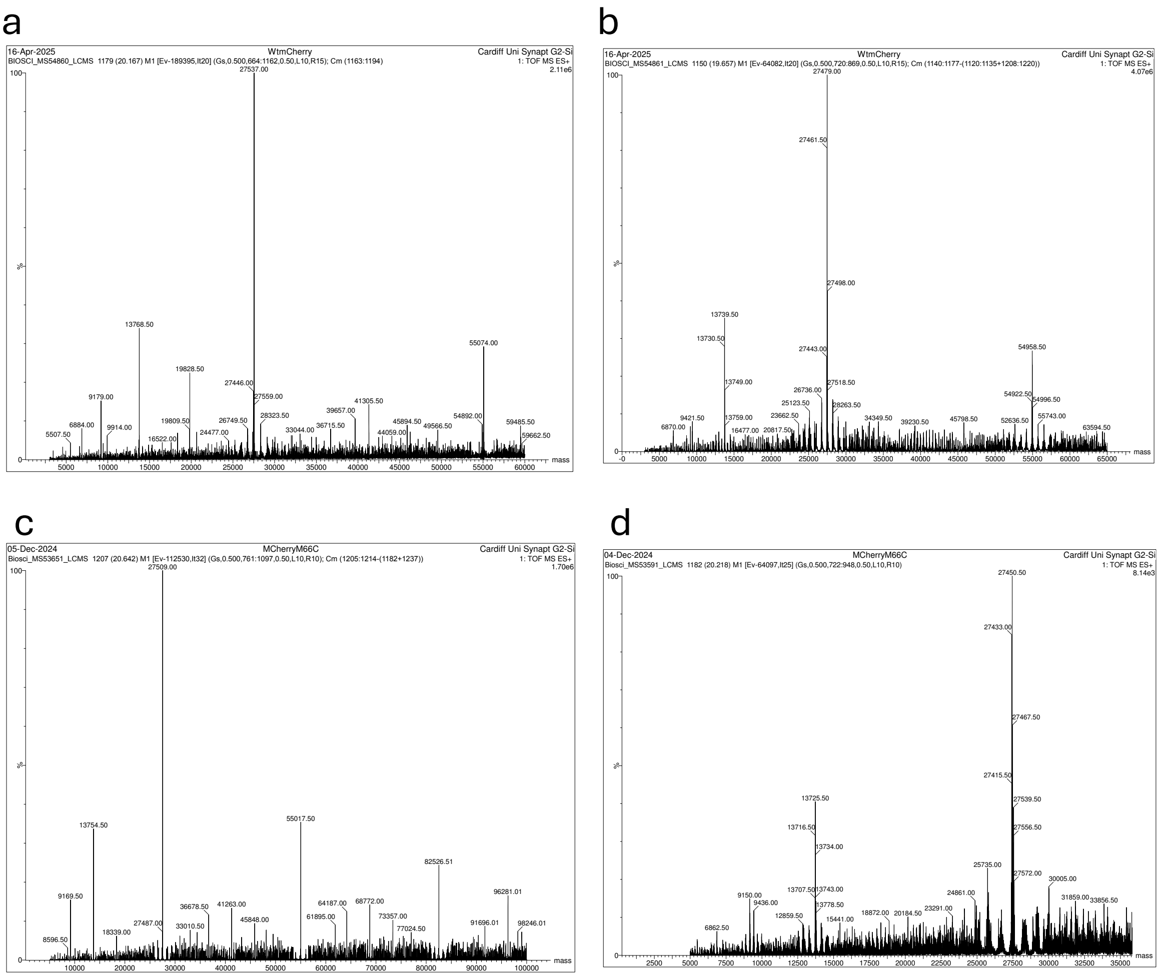

Supplementary Figure S3. Mass spectra of mCherry before (a) and after (d) addition of 0.1 % (v/v) H_2O_2, and of mCoral before (c) and after (d) addition of 0.1 % (v/v) H_2O_2. The main peak values are: (a) 27537.0 Da; (b) 27479.0 Da; (c) 27509.0 Da; (d) 27450.5 Da. Comparison with predicted protein masses are shown in the main manuscript (Figure 3e).

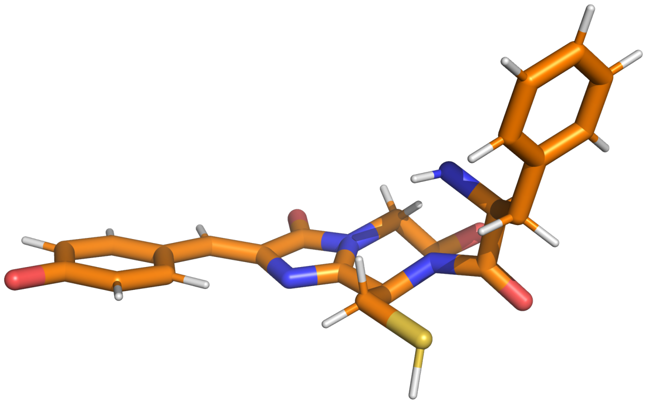

Supplementary Figure S4. The mCoral chromophore unit used in MD simulations.

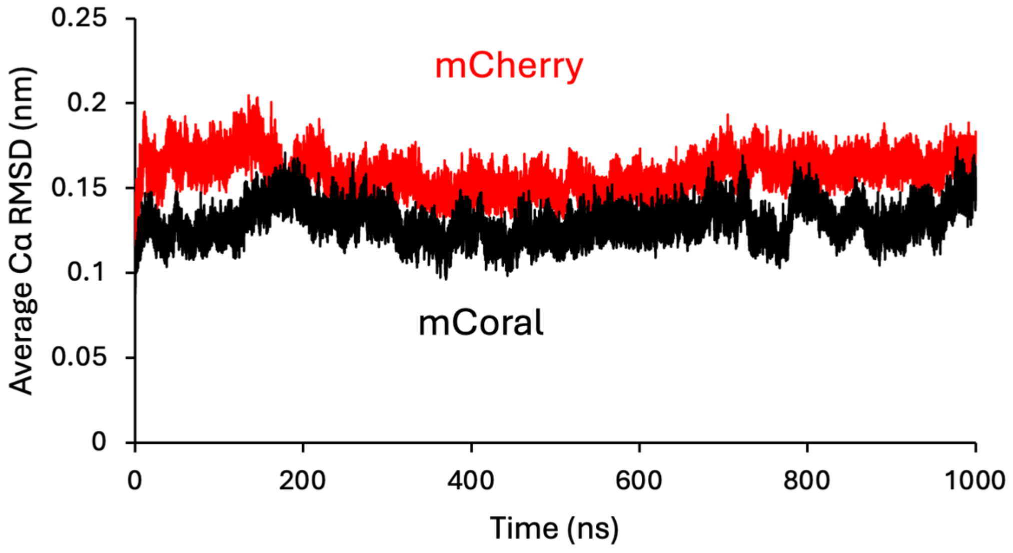

Supplementary Figure S5. Average Cα RMSD over 3 x 1000 ns MD runs for mCherry (red) and mCoral (black).

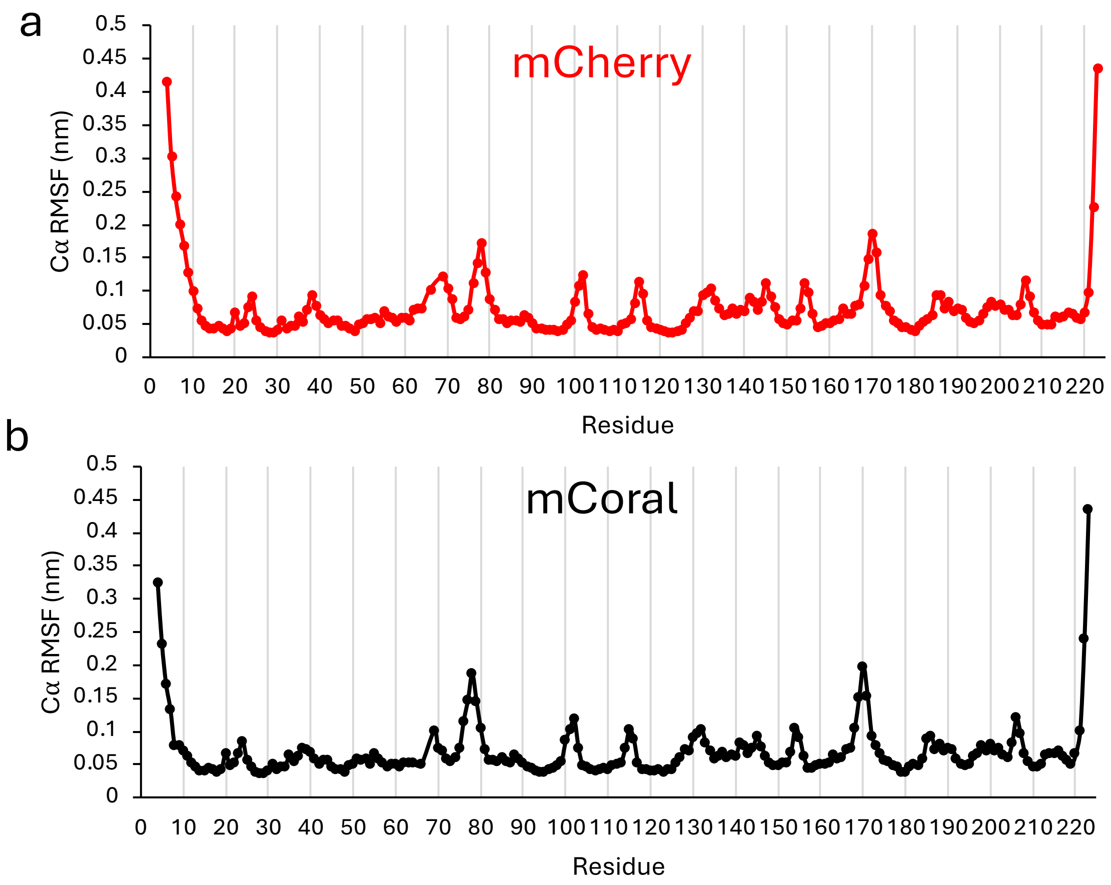

Supplementary Figure S6. Average Cα RMSF over 3 x 1000 ns MD runs for (a) mCherry and (b) mCoral.

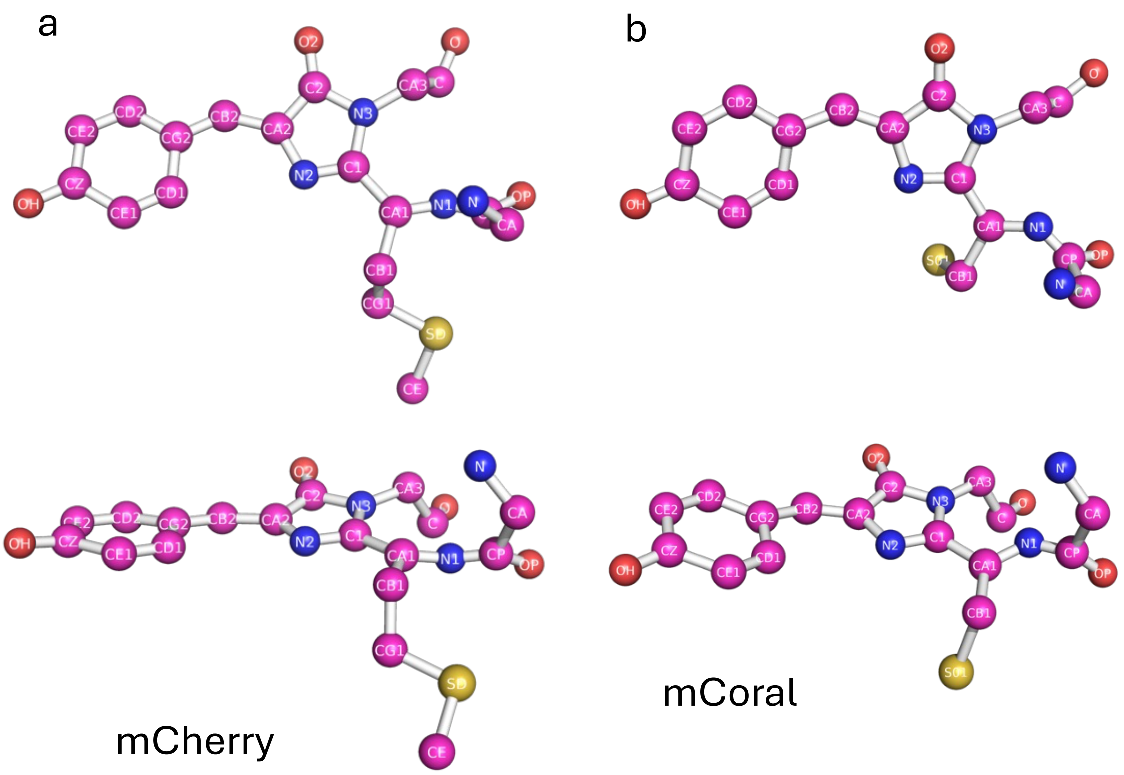

Supplementary Figure S7. CRO atom nomenclature for (a) mCherry and (b) mCoral.

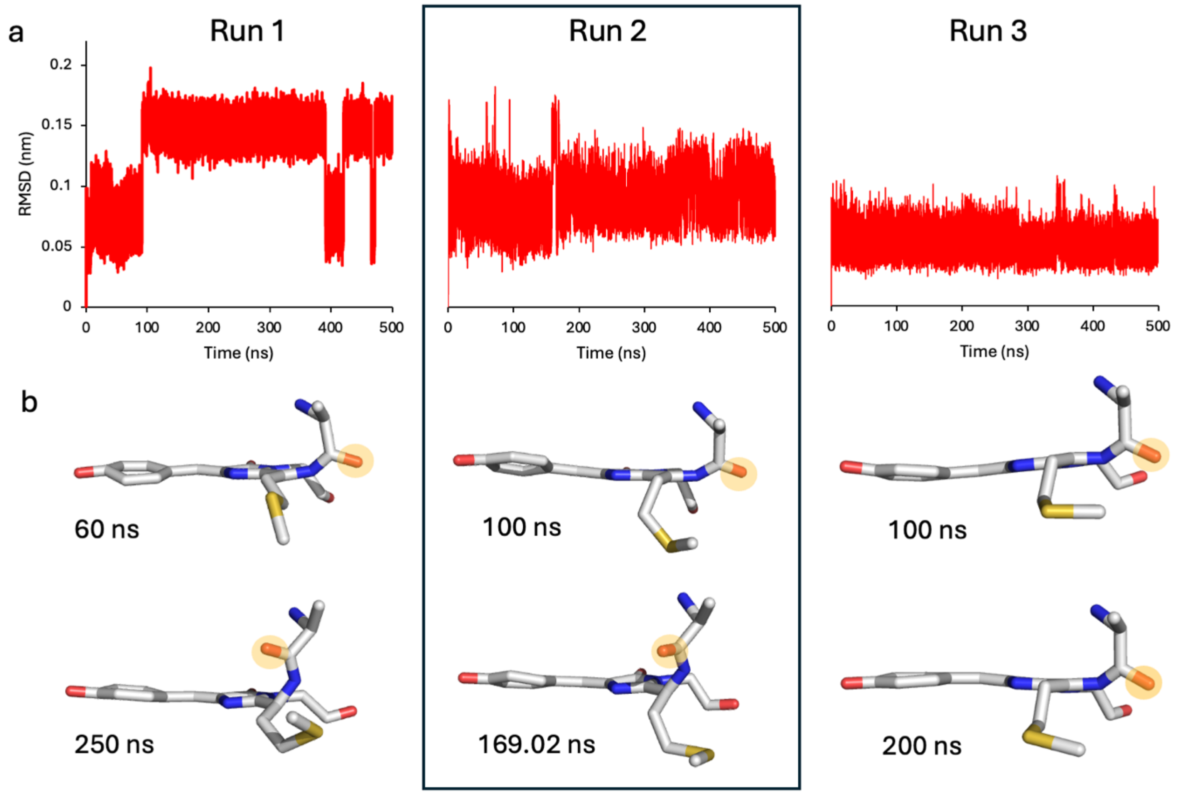

Supplementary Figure S8. MD simulations of mCherry with de novo added water. (a) The CRO (all atoms) RMSD profile over the 3 runs. (b) Different chromophore configurations of mCherry extracted from the 500 ns simulations at time points outlined on the figure. The orange circle highlights the F65 carbonyl oxygen.
